# Microfluidics: A New Layer of Control for Extrusion-Based 3D Printing

**DOI:** 10.3390/mi9020086

**Published:** 2018-02-16

**Authors:** Ludovic Serex, Arnaud Bertsch, Philippe Renaud

**Affiliations:** EPFL STI IMT LMIS4, Station 17, CH-1015 Lausanne, Switzerland; arnaud.bertsch@epfl.ch (A.B.); philippe.renaud@epfl.ch (P.R.)

**Keywords:** micro-fluidic, additive manufacturing, 3D printing, bio-printing, lab on a tip

## Abstract

Advances in 3D printing have enabled the use of this technology in a growing number of fields, and have started to spark the interest of biologists. Having the particularity of being cell friendly and allowing multimaterial deposition, extrusion-based 3D printing has been shown to be the method of choice for bioprinting. However as biologically relevant constructs often need to be of high resolution and high complexity, new methods are needed, to provide an improved level of control on the deposited biomaterials. In this paper, we demonstrate how microfluidics can be used to add functions to extrusion 3D printers, which widens their field of application. Micromixers can be added to print heads to perform the last-second mixing of multiple components just before resin dispensing, which can be used for the deposition of new polymeric or composite materials, as well as for bioprinting new materials with tailored properties. The integration of micro-concentrators in the print heads allows a significant increase in cell concentration in bioprinting. The addition of rapid microfluidic switching as well as resolution increase through flow focusing are also demonstrated. Those elementary implementations of microfluidic functions for 3D printing pave the way for more complex applications enabling new prospects in 3D printing.

## 1. Introduction

Three-dimensional (3D) printing, also commonly referred to as additive manufacturing or rapid prototyping, is a set of techniques that consist in building 3D parts layer by layer. This fabrication principle dates from the early 1980s [1] and has seen a number of different implementations based on the use of multiple deposition techniques [2]. While photopolymers and thermoplastic polymers were initially used in 3D printing techniques, the choice of materials that can be used has been significantly widened, and includes metals [3,4], ceramics [5] and biomaterials [6,7,8,9,10,11]. Current research in this field includes the development of “smart materials” [12,13] that can evolve with time and bring additional functions to the fabricated objects. If 3D printing was first used for automotive and aerospace applications [14,15], many other application fields currently use these techniques, including medical application [16,17,18,19], tissue enginering [20,21,22,23,24], biosensors [25], microfabrication [26,27] or even construction [28] and the food industry [29]. With hobbyists now having access to 3D printing, it is likely that its field of applications will expand even more. 

Among these 3D printing methods, stereolithography (SLA) and extrusion based system dominate the market. SLA is known for its very high resolution [30], but is subject to limitations directly related to the process itself, such as the limited biocompatibility of the materials that can be used (often linked to the use of photoinitiators) and the very challenging implementation of multi-material printing machines. On the other hand, extrusion-based processes are increasingly popular [31] as they are relatively cheap and easy to use. However, this printing technique suffers from its limited number of printable materials: only low melting temperature materials such as ABS for fused deposition modeling or fast crosslinking materials for bio-printing can be used. Additionally, in recent years, the need for smarter dispensing tools has emerged, in particular in the field of bioprinting to answer the need to print complex materials for cells [32,33,34]. As the field increasingly aims toward regenerative medicine [9,35,36,37]. A few implementations of such smart dispensing tools have been already presented in the literature, such as print heads made from needles used for manufacturing perfusable vascular constructs [38]. To further overcome this limitation, extrusion-based 3D printing can benefit from more complex microfluidic systems, which can implement a number of fluidic manipulation functions at the micro-scale. Microfluidics has seen major developments in recent years, and has contributed to the emergence of the concept of “Lab on a chip” by allowing the implementation of many fluidic functions such as micro-mixers [39,40,41], switching valve [42,43,44], flow focusing [45], particles focusing [46,47,48], in-channel detection [49] or particles and cell sorting [49,50,51,52] in compact, microfabricated devices.

Up until now, these functionalities have mainly been used on chip to perform various analysis. In this paper, we propose to exploit the potential of microfluidics to develop a “lab on a tip” that could perform various operations on the dispensing solution directly in the print head of the 3D printer. Using this principle, we demonstrate multiple smart printing heads that allow the use of new materials, enhance the print resolution, or allow the printing of composite parts or multi-material parts that were only possible using expensive 3D printing techniques.

## 2. Materials and Methods 

### 2.1. Probe Fabrication

All the print head dispensing tips that are presented in this paper were manufacture in a similar way using specific microchannel designs depending on the targeted application. A 500 nm SiO_2_ layer is first grown by wet oxidation on a single-side polished silicon wafer. Standard photolithography is performed to pattern the fluidic channels and the silicon oxide is etched to create a hard mask. In the case of the herringbone mixer presented in Section 3.3, which requires a two-level microchannel to be manufactured, an additional photoresist mask, silicon etching and resist striping is performed as described in the previous literature [39]. Then, the channels are etched to 300 µm in depth using deep reactive ion etching, and the top-layer oxide is etched away as well to expose the silicon for anodic bonding. Next, a borofloat^®^ 33 glass wafer is mechanically drilled to create the probes inlets. The two wafers are cleaned with a piranha solution and bonded by anodic bonding. The wafer stack is then diced, revealing the dispensing outlet in a similar fashion as described in the previous literature [53].

### 2.2. 3D Printer Setup

The printer setup is similar to the one presented in previous work [53]. A DLT-180, Double Delta 3D printer has been purchased from He3D and adapted to fit the fabricated probes. The printing path and speed was either hard-coded in G-code or automatically generated using a slicer as is usually done for standard 3D printing.

## 3. Results

In this section we present four different probes that can perform specific tasks. Those probes demonstrate how microfluidics can be implemented to improve extrusion-based 3D printing in terms of printed materials, resolution or material switching.

### 3.1. Multi-Material

One limitation of extrusion-based 3D printers is that each print head can only print a single material. Printing an object made of multiple materials is usually achieved by using a 3D printing machine with multiple extrusion heads placed next to each other. By switching between print heads, different materials can then be printed [54]. In addition to requiring specific equipment, printing multi-material components with extrusion-based 3D printers is generally a very slow process, and a smooth transition between printed materials is not always guaranteed. A number of microfluidic systems allowing switching between different liquids have already been presented in the literature, including some that could be implemented in 3D printers [44]. Here, we will show a very simple system that is easy to implement and allows a fast switching between materials.

**Figure 1:**
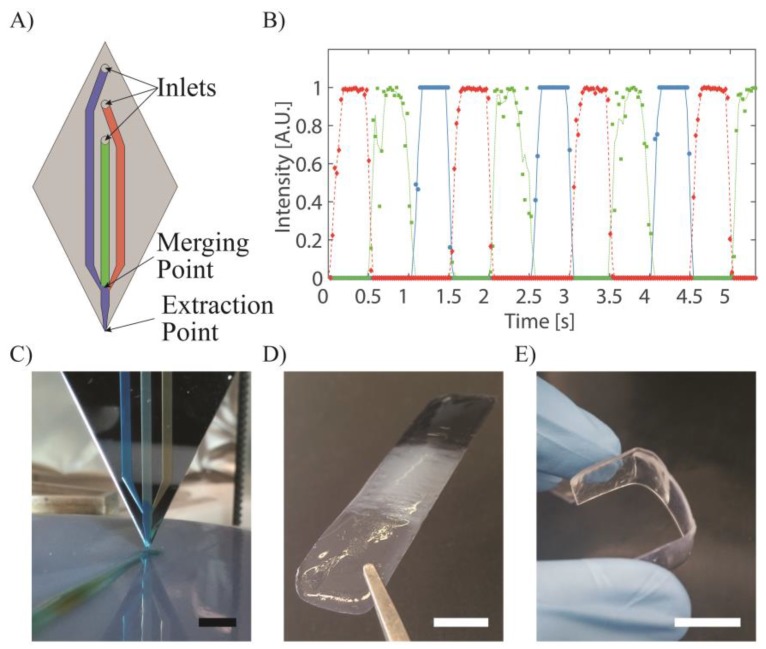
Multi-channel print head. (**A**) Schematic of the microfluidic channel design. (**B**,**C**) Switching of three colored liquids as recorded at the tip of the print head. (**D**) Clear, white and black part. (**E**) Two-hinges part printed with two different inks (one rigid and one flexible). All scale bars correspond to 1 cm.

The print head we designed is composed of three micro-channels merging into one right before the ejection point, as presented in Figure 1A. Each of these three channels can be connected to a syringe containing a different material and actuated by a syringe pump. By choosing the sequence of actuation of the syringe pumps and synchronizing it with the geometry of the part being built, a seamless transition between multiple materials can be achieved during the object manufacturing process. Furthermore, as microfluidic benefits from very small dead volumes, it is possible to rapidly switch between materials. In our case, a complete transition between two materials can be performed in 500 ms, as presented in Figure 1B, where the transition between different colored liquids was recorded using a camera connected to a microscope focused at the tip of the print head we used. 

The print head was then mounted on a 3D printer, and colored alginate solutions were printed on an agarose 4% and CaCl_2_ 1% bed, inducing the alginate gelation by diffusion of the calcium ions into the alginate deposit. Figure 1C shows the smooth transition between colors obtained as the printing process goes. 

By combining extrusion 3D printing with ultraviolet (UV) light irradiation of the printed layers, it is possible to print and crosslink photosensitive inks using the same print head based on merging microchannels. Thus, multimaterial components can be made by the extrusion method by selecting photosensitive resins having the desired properties, chosen from the large catalogue of materials developed for the stereolithography process. As an example, we used Formlabs resins RS-F2-GPCL-04 (which results in a transparent and rigid material once cured), RS-F2-GPWH-04 (which is a white resin) and RS-F2-GPBK-04 (which is a black resin). By switching between these resins, we were able to print multicolor parts, as shown in Figure 1D. Formlabs also developed a flexible resin (RS-F2-GPGR-02). By alternating rigid and flexible areas, we can create parts with varying Young’s moduli. In this example, a two-hinge part was printed (Figure 1E).

Switching between two different materials can be performed in a cleaner and faster way that the one used in the very simple multi-channel print head presented in Figure 1, for example by using actuated virtual valves similar to the ones presented by Braschler et al. [42]; however, this method induces the waste of larger amounts of printing material, which can be a problem. 

In the examples we presented, the materials were deposited alternately one after the other, using microfluidic switching, but multiplexing can also be implemented directly in the print head, allowing the alternation of materials both laterally and during printing. Microfluidic devices capable of performing such types of multiplexing have been demonstrated in Lab on Chips, using polydimethylsiloxane (PDMS) as structural material, as it allows a simple fabrication and actuation of valves, but its use in print heads for 3D printing may be limited, as the flexibility of PDMS would limit the extrusion pressure [55]. 

### 3.2. Enhanced Resolution

**Figure 2:**
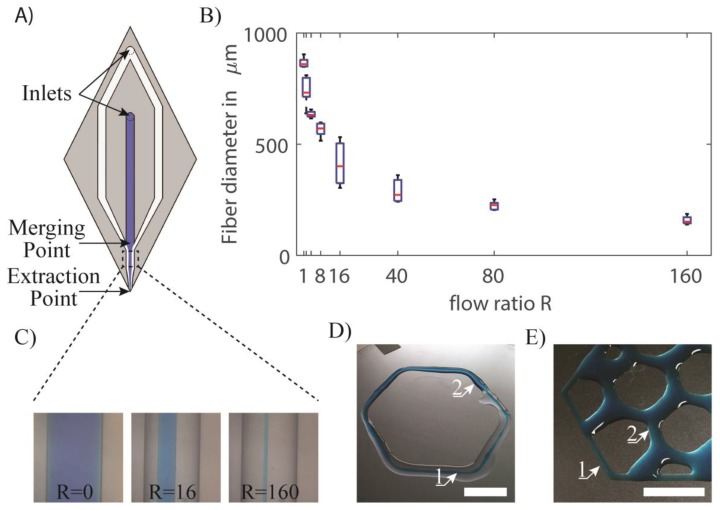
Print head based on flow focusing. (**A**) Principle of hydrodynamic flow focusing with the core flow in blue and the sheath flow in white. (**B**) Flow focusing at different sheath flow/core flow ratios (R). (**C**) Printed fiber diameter as a function of the flow ratio. (**D**) Printed filament going from small filament (1) to large filament (2) whilst the ratio R is changed. (**E**) First layer of a printed part with fine edge (1) and coarse filling (2). Scale bars = 5 mm.

Microfluidic flows present a laminar behavior [45]. This property can be exploited to change the resolution of the printed material by focusing it while it is dispensed using sheath flows [25]. Hydrodynamic focusing only requires a very rudimentary microfluidic setup, the most common configuration being a 3-channel device, where the center flow stream is pinched between two side flow streams, resulting in a shrinking of the width of the center flow. Implementing hydrodynamic focusing in the print head of an extrusion-based 3D printer, its resolution can be not only be significantly increased, but it can be adjusted while printing by simply varying the ratio between the core and lateral flows. Figure 2A demonstrates the principle of sheath flow. Alginate filaments (in blue) are focused using de-ionized (DI) water with 4% CaCl_2_. Depending on the ratio of the alginate core flow to the CaCl_2_ solution sheath flow (Figure 2B), the obtained filaments can vary in diameter from 800 µm to below 200 µm, providing a fivefold resolution increase. Figure 2C shows the evolution of the size of the deposited filaments as a function of the ratio between sheath and core flows.

One issue of the use of sheath flows for increasing the printing resolution is the fact that the liquid used in the sheath flow for focusing is also “printed” alongside the actual material of interest. In our example, using a water-based solution, large quantities of water are ejected from the probe and may disrupt the printing. On Figure 2D, a printed filament with changing diameter is presented. The first section of the filament is small and soaked in the sheath flow that will ultimately dry. The filament then widens and the surplus of liquid produced by the sheath flow becomes almost nonexistent as it is reduced to a minimum. To solve the problem of residual fluid, Perfluoro(methyldecalin) (PFD) was used as sheath flow. Given that it is a highly volatile material, it will quickly evaporate, leaving only the desired focused filament. One limitation of this technique is that when the sheath flow becomes much larger than the core flow, flow instabilities occur, limiting the use of hydrodynamic focusing for enhancing the resolution of the dispensed material. In this case, there is always the possibility of reducing the dimensions of the microfabricated channels provided on the print head for improving the resolution. However, this implies the use of larger values of pressure to drive the various liquids in the microchannels. Furthermore, it is important to note that as liquids are being printed, the tuning of the printed filament through flux control of the core flow only without flow focusing can be achieved. With a constant printing speed, an increase of the flux will result in a larger printed filament (Figure 2E). This presents the advantage of not having residual liquid from the sheath flow, but the crosslinking must be performed from the probe either by UV light or, in this case, by printing alginate on a CaCl_2_ bed, which is not suitable for high structures.

Adjusting the printer resolution on the fly opens new ways of controlling the printed part. It would thus be possible to choose the resolution depending on the importance of the part being printed while printing. This methods allows the printing of coarse filament for the filling of the printed part, while at the same time, for the wall, the filament can be refined to obtain fast and precise printing.

### 3.3. New Materials

Laminar flows have the particularity that they do not mix easily, as turbulence does not occur in this flow regime. Mixing relies solely on the diffusion phenomenon, which can be enhanced by splitting and rearranging the flow using a mixer. Many micro-mixers [41] have been devised for microfluidic applications; of these static micro-mixers have the advantage of being passive structures that provide efficient mixing in laminar conditions and that can be integrated directly in the print head nozzles used for 3D printing. 

In a previous work, we demonstrated the use of a meander-based static micro-mixer for performing the mixing of slow reacting materials used to 3D print carboxymethylcellulose-based cryogels [53]. A micro-fabricated dispensing print head allowing both the last-second mixing of cryogel precursors and the temperature control of the extruded material during printing was used. This allowed the 3D printing of multi-layer cryogels with on-demand local pore size change through the control in temperature of the dispensed solution. The seeding and culture of cells in specific areas of the obtained 3D cryogel structure was also demonstrated after its functionalization.

**Figure 3:**
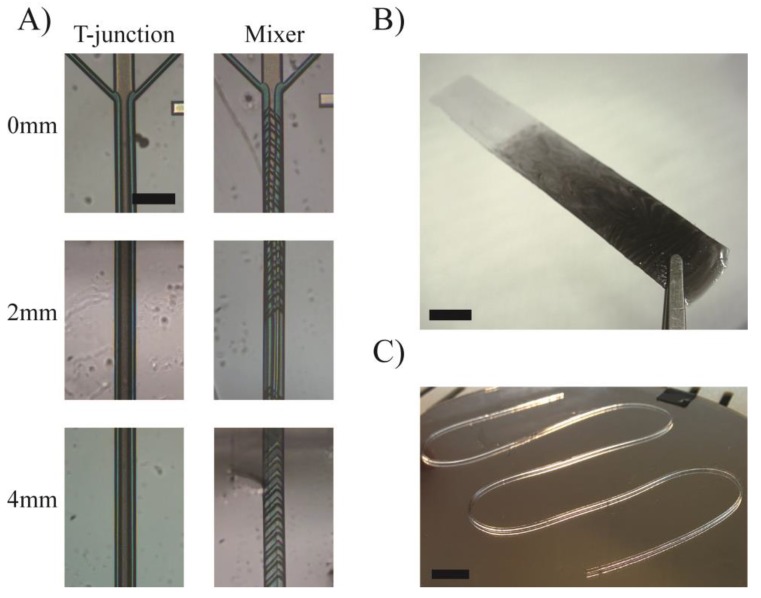
Herrigbone micro-mixer integrated at the tip of the probe. (**A**) Colored glycerol streams pushed through a simple T-junction and through a herringbone micro-mixer at 0 mm, 2 mm and 4 mm after the merging point. The mixer allows rapid and efficient mixing of viscous materials. Scale bar = 300 µm. (**B**) Mixing of white and black Formlabs resins at different ratios results in smooth gradients from one material to the other Scale bar = 1 cm. (**C**) Printed acrylate filament made using a two-component material. Scale bar = 1 cm.

There are, however, static mixers much more efficient than meander-based micro-mixers. Herrigbone mixers [39] still remain to this day among the most efficient static micro-mixers, but their manufacturing using microfabrication technologies is slightly more complicated than that of meander-based mixers, as they are two-level structures and require a larger number of fabrication steps. By implementing herringbone micro-mixers at the tip of a dispensing print head, even viscous materials can be mixed in structures of only a few millimeters in length. This principle is demonstrated in Figure 3A, where colored glycerol streams (in blue and white) are pushed through two three-inlet devices, one being a simple straight microchannel, the other accommodating a herringbone micro-mixer. Mixing occurs within the first 5 mm of the herringbone structure, whereas there is no apparent mixing in the straight channel. Because different materials can be efficiently mixed, gradients can be created by variating their ratio. As an example, black and white Formlabs resins were printed going from fully black to fully white in a smooth gradient (Figure 3B). Therefore by adding the 3 primary colors to the mix, any color can be printed in a very simple manner. 

Moreover, as mixing is performed, fast-reacting material can be brought in contact, just before they are deposited by 3D printing. As a demonstration, we performed the mixing of a two-component polymerizable resin based on polyethylene glycol 400 diacrylate, one component containing 3% N,N,N’,N’ Tetramethyl-ethylenediamine, the other containing 3% benzoyl peroxide. The benzoyl peroxide/amine system is known as an initiator for free-radical polymerization of acrylates [56] and the reaction is fast when all materials are brought in contact. By adequately choosing the amine component, the crosslinking speed can be adapted. Figure 3C shows a 3D printed filament made by mixing these components at the last second.

The use of micro-mixers placed at the output of the print head of extrusion based 3D printers allows to mix reactive components right before dispensing them. This allows materials that are not usually used in 3D printing to be printed, such as polymer systems initiated by peroxides, as well as biomaterials such as hydrogels or cryogels. Additionally, more conventional materials can be mixed with a chosen ratio between them, allowing blends of materials to be created. This is of interest in the production of materials with gradients of properties in an object. It can simply be a gradient of colors if two resins of different color are used, but gradients of mechanical properties can also be fabricated, for example by mixing resins resulting in flexible and hard polymers or by mixing composite resins, charged with a filler material.

### 3.4. Concentration

**Figure 4:**
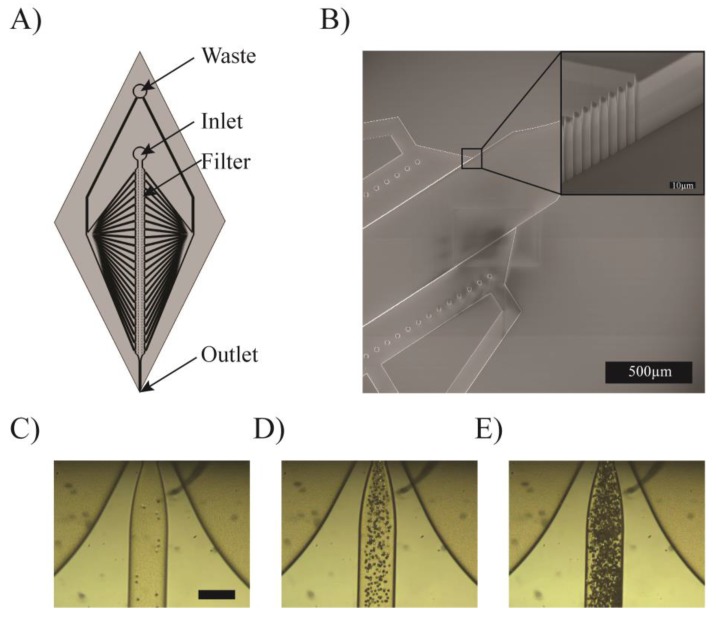
Crossflow filter for particle concentration. (**A**) Schematic of the print head. (**B**) Scanning electron micrograph of the crossflow filter. (**C**–**E**) Concentration of 8µm beads by adjusting the withdrawing factor. Scale bar = 250 µm.

In the previous paragraphs, print heads based on very simple microfluidic components were presented; however, microfabrication techniques can be used to manufacture much more complex structures. In this paragraph, we demonstrate a print head allowing the concentration of particles in solution, which can be used to increase the filler concentration of a composite resin just before it is printed or to concentrate cells in bioprinting. Figure 4A shows a schematic of the print head. A solution containing particles or cells enters the device by the inlet and flows in a straight channel equipped with a crossflow filter towards the outlet. This crossflow filter allows some of the liquid to be withdrawn between inlet and outlet simply by adequately imposing the flows both at the inlet and the waste outlet, thus concentrating the particles in solution. The extracted liquid is removed from the device through the waste outlet, while the concentrated solution is 3D printed. To separate micrometric-sized particles, the crossflow filter must feature small openings. This is made by a series of 50 µm tall, 2 µm wide pillars with a 4 µm pitch, as shown in Figure 4B. This structure is fabricated in a single etching step. Figure 4C–E demonstrates the concentration of a solution containing 8 µm beads.

Concentrating particles in solution before their being dispensed by 3D printing can find applications in various fields. It is known that the concentration of particles in composite materials is a major factor in their physical properties [57]. With the device we presented, the physical properties of the printed composites could be changed during printing. This is of particular interest when high concentrations of filler material need to be present in the printed material, making it very viscous. Concentrating the particles in a composite material at the last second when printing makes it possible to work with diluted (thus of low viscosity) solutions for the whole process and still produce composites of the desired composition, removing the excess liquid just before dispensation.

Concentrating cells in solution is of even bigger interest in the field of bio-printing, as intercellular communications rely heavily on intercellular distance [58], and thus on the cell concentration. However, working with high concentrations of cells in bioprinting is often inappropriate, as a large quantity of cells would be lost in pipes and other dead volumes, and the cells would be subjected to large shear forces because of the high viscosity of the solution. In general bioprinting techniques use solutions containing roughly 1 million cells per milliliter of solution, which results in cell-laden gels containing a cell concentration orders of magnitude lower than in native tissue [56].

By concentrating the cell right at the tip of the printer, it is possible to print tissue with biologically relevant cell concentration, without wasting large numbers of cells in dead volumes or subjecting them to high shear stress.

## 4. Discussion

Stereolithography and extrusion-based 3D printing are the most commonly used additive manufacturing techniques. Extrusion-based techniques are easy to use and are cost effective, but have seen almost no improvement in terms of process development since their creation. On the contrary, microfluidic techniques have seen major developments in terms of device design and fabrication, and these developments can now be applied to significantly improve the print heads of extrusion-based 3D printers.

In this paper, we explored the integration of four relatively simple microfluidic systems at the tip of the print head of extrusion-based 3D printers to create smart dispensing tools that can perform different functions right before printing. The integration of additional functions to print heads is of particular importance for bioprinting applications. We demonstrated fast switching between two or more materials to create multimaterial parts, but when applied to bioprinting, it can be used to switch between the different cell types needed in a cell co-culture. Enhancing the printing resolution using sheath flows can also be used for centering cells inside hydrogel filaments. Micro-mixers can be used to prepare hydrogels or cryogels from their components, to insert cells into the printing medium or to create gradients of composition or of cell types while printing. Concentration using crossflow filters makes it possible to reach cell concentrations close to native tissue without adverse effects during their manipulation.

Thanks to microfabrication, more complex functions can also be integrated in the print heads, taking advantage of all the knowledge available in the field of Lab on Chip devices, but such developments would probably have precise needs in terms of specific applications. For example, combining fluidic functions with in-channel spectroscopy could provide continuous feedback on the printed material quality, implementing dielectrophoresis electrodes could be used for filtering particles and cells and could make it possible to print only the viable cells, and detecting the passage of cells of interest while they flow through the microchannels and filtering out the excess of liquid medium could make it possible to print each cell at a precisely chosen location. Many other microfluidic functions can be integrated, such as fast switching valves, digital microfluidic, microfluidic gradient generators, cell concentrator or micro-heaters, each bringing the potential of using new materials and developing new applications for extrusion-based 3D printing.

The integration of customized microfluidic functions into the print heads of 3D printers has the potential to be a game changer in fields where additive manufacturing is used, and in particular for bioprinting. Compared to more conventional 3D printing, there is an additional level of complexity involved when making biological constructs out of different types of cells and scaffold materials, and the use of cells in bioprinting applications requires more care than the usual materials used in additive manufacturing techniques. Bioprinting is often described as the key development to go towards artificial organs and engineered biological tissues, but to obtain the level of complexity needed to form such complex cell constructs, major improvements in 3D printing techniques are needed, and one of the keys to achieving this is clearly the integration of microfluidic functions into print heads.

## 5. Conclusions

New opportunities generally arise when merging different technologies, and the present paper attempts to show the value of bringing microfluidics closer to extrusion-based 3D printing techniques. By applying some concepts developed over the years for Lab on Chip applications to create smart print heads, we tried to open the way and demonstrate improved dispensing functionalities. Four different microfluidic functions were implemented at the tip of print heads: Micro-mixers were used to mix reactive components before dispensing and to blend materials with varying ratios. Sheath flows were implemented to improve the print resolution. Fast switching was demonstrated to create multimaterial components and crossflow filtration was shown to concentrate particles or cells in the deposited material. By using microfluidic functions in the world of 3D printing, an additional layer of control can be added, enabling the fabrication of components that were impossible to print before. This methodology can be scaled, and has the potential to revolutionize the way we print, in particular in the field of bioprinting, where new challenges need to be faced in going towards the fabrication of engineered 3D biological tissues.

## References

[1] Kodama H. (1981). Automatic method for fabricating a threedimensional plastic model with photo hardening polymer. Rev. Sci. Instrum..

[2] Gibson I., Rosen D., Stucker B. (2015). Additive Manufacturing Technologies. Additive Manufacturing Technologies.

[3] Todd I. (2017). Metallurgy: Printing steels. Nat. Mater..

[4] Sames W.J., List F.A., Pannala S., Dehoff R.R., Babu S.S. (2016). The metallurgy and processing science of metal additive manufacturing. Int. Mater. Rev..

[5] Eckel Z.C., Zhou C., Martin J.H., Jacobsen A.J., Carter W.B., Schaedler T.A. (2016). Additive manufacturing of polymer-derived ceramics. Science.

[6] Hockaday L.A., Kang K.H., Colangelo N.W., Cheung P.Y.C., Duan B., Malone E., Wu J., Girardi L.N., Bonassar L.J., Lipson H. (2012). Rapid 3D printing of anatomically accurate and mechanically heterogeneous aortic valve hydrogel scaffolds. Biofabrication.

[7] Jang J., Park J.Y., Gao G., Cho D.W. (2018). Biomaterials-based 3D cell printing for next-generation therapeutics and diagnostics. Biomaterials.

[8] Murphy S.V., Atala A. (2014). 3D bioprinting of tissues and organs. Nat. Biotechnol..

[9] Hospodiuk M., Dey M., Sosnoski D., Ozbolat I.T. (2017). The bioink: A comprehensive review on bioprintable materials. Biotechnol. Adv..

[10] Kolesky D.B., Homan K.A., Skylar-Scott M.A., Lewis J.A. (2016). Three-dimensional bioprinting of thick vascularized tissues. Proc. Natl. Acad. Sci. USA.

[11] Pataky K., Braschler T., Negro A., Renaud P., Lutolf M.P., Brugger J. (2012). Microdrop Printing of Hydrogel Bioinks into 3D Tissue-Like Geometries. Adv. Mater..

[12] Sydney Gladman A., Matsumoto E.A., Nuzzo R.G., Mahadevan L., Lewis J.A. (2016). Biomimetic 4D printing. Nat. Mater..

[13] Khoo Z.X., Teoh J.E.M., Liu Y., Chua C.K., Yang S., An J., Leong K.F., Yeong W.Y. (2015). 3D printing of smart materials: A review on recent progresses in 4D printing. Virtual Phys. Prototyp..

[14] Guo N., Leu M.C. (2013). Additive manufacturing: Technology, applications and research needs. Front. Mech. Eng..

[15] Chu M.Q., Wang L., Ding H.Y., Sun Z.G. (2015). Additive Manufacturing for Aerospace Application. Appl. Mech. Mater..

[16] Prasad L.K., Smyth H. (2016). 3D Printing technologies for drug delivery: A review. Drug Dev. Ind. Pharm..

[17] Bose S., Ke D., Sahasrabudhe H., Bandyopadhyay A. (2017). Additive Manufacturing of Biomaterials. Prog. Mater. Sci..

[18] Davia-Aracil M., Hinojo-Pérez J.J., Jimeno-Morenilla A., Mora-Mora H. (2018). 3D printing of functional anatomical insoles. Comput. Ind..

[19] Rengier F., Mehndiratta A., Von Tengg-Kobligk H., Zechmann C.M., Unterhinninghofen R., Kauczor H.U., Giesel F.L. (2010). 3D printing based on imaging data: Review of medical applications. Int. J. Comput. Assist. Radiol. Surg..

[20] Zhang Y.S., Yue K., Aleman J., Mollazadeh-Moghaddam K., Bakht S.M., Yang J., Jia W., Dell’Erba V., Assawes P., Shin S.R. (2017). 3D Bioprinting for Tissue and Organ Fabrication. Ann. Biomed. Eng..

[21] Wu Z., Su X., Xu Y., Kong B., Sun W., Mi S. (2016). Bioprinting three-dimensional cell-laden tissue constructs with controllable degradation. Sci. Rep..

[22] Dvir-Ginzberg M., Gamlieli-Bonshtein I., Agbaria R., Cohen S. (2003). Liver tissue engineering within alginate scaffolds: Effects of cell-seeding density on hepatocyte viability, morphology, and function. Tissue Eng..

[23] Zadpoor A.A., Malda J. (2017). Additive Manufacturing of Biomaterials, Tissues, and Organs. Ann. Biomed. Eng..

[24] Roth E.A., Xu T., Das M., Gregory C., Hickman J.J., Boland T. (2004). Inkjet printing for high-throughput cell patterning. Biomaterials.

[25] Lind J.U., Busbee T.A., Valentine A.D., Pasqualini F.S., Yuan H., Yadid M., Park S.J., Kotikian A., Nesmith A.P., Campbell P.H. (2017). Instrumented cardiac microphysiological devices via multimaterial three-dimensional printing. Nat. Mater..

[26] Vaezi M., Seitz H., Yang S. (2013). A review on 3D micro-additive manufacturing technologies. Int. J. Adv. Manuf. Technol..

[27] Au A.K., Huynh W., Horowitz L.F., Folch A. (2016). 3D-Printed Microfluidics. Angew. Chem. Int. Ed..

[28] Salet T.A.M., Bos F.P., Wolfs R.J.M., Ahmed Z.Y. 3D concrete printing—A structural engineering perspective. Proceedings of the 2017 fib Symposium—High Tech Concrete: Where Technology and Engineering Meet.

[29] Kouzani A.Z., Adams S., Oliver R., Nguwi Y.Y., Hemsley B., Balandin S. 3D printing of a pavlova. Proceedings of the Region 10 Conference (TENCON).

[30] Bertsch A., Bernhard P., Vogt C., Renaud P. (2000). Rapid prototyping of small size objects. Rapid Prototyp. J..

[31] Turner B.N., Strong R., Gold S.A. (2014). A review of melt extrusion additive manufacturing processes: I. Process design and modeling. Rapid Prototyp. J..

[32] Liu W., Zhang Y.S., Heinrich M.A., De Ferrari F., Jang H.L., Bakht S.M., Alvarez M.M., Yang J., Li Y.C., Trujillo-de Santiago G. (2017). Rapid Continuous Multimaterial Extrusion Bioprinting. Adv. Mater..

[33] Hardin J.O., Ober T.J., Valentine A.D., Lewis J.A. (2015). Microfluidic printheads for multimaterial 3D printing of viscoelastic inks. Adv. Mater..

[34] Nie M., Mistry P., Yang J., Takeuchi S. Microfluidic enabled rapid bioprinting of hydrogel μfiber based porous constructs. Proceedings of the 2017 IEEE 30th International Conference on Micro Electro Mechanical Systems (MEMS).

[35] Tan X.P., Tan Y.J., Chow C.S.L., Tor S.B., Yeong W.Y. (2017). Metallic powder-bed based 3D printing of cellular scaffolds for orthopaedic implants: A state-of-the-art review on manufacturing, topological design, mechanical properties and biocompatibility. Mater. Sci. Eng. C.

[36] Yu Y., Moncal K.K., Li J., Peng W., Rivero I., Martin J.A., Ozbolat I.T. (2016). Three-dimensional bioprinting using self-Assembling scalable scaffold-free ‘tissue strands’ as a new bioink. Sci. Rep..

[37] Duan B. (2017). State-of-the-Art Review of 3D Bioprinting for Cardiovascular Tissue Engineering. Ann. Biomed. Eng..

[38] Jia W., Gungor-Ozkerim P.S., Zhang Y.S., Yue K., Zhu K., Liu W., Pi Q., Byambaa B., Dokmeci M.R., Shin S.R. (2016). Direct 3D bioprinting of perfusable vascular constructs using a blend bioink. Biomaterials.

[39] Stroock A.D., Dertinger S.K., Ajdari A., Mezić I., Stone H.A., Whitesides G.M. (2002). Chaotic mixer for microchannels. Science.

[40] Liu Y.Z., Kim B.J., Sung H.J. (2004). Two-fluid mixing in a microchannel. Int. J. Heat Fluid Flow.

[41] Lee C.Y., Chang C.L., Wang Y.N., Fu L.M. (2011). Microfluidic Mixing: A Review. Int. J. Mol. Sci..

[42] Braschler T., Theytaz J., Zvitov-Marabi R., Van Lintel H., Loche G., Kunze A., Demierre N., Tornay R., Schlund M., Renaud P. (2007). A virtual valve for smooth contamination-free flow switching. Lab Chip.

[43] Liepsch D., Versorgungstechnik F., Germany W., Karlsruhe U., Vlachos N.S. (1982). Measurement and calculations of laminar in A ninety degree bifurcation. J. Biomech..

[44] Devaraju N.S.G.K., Unger M.A. (2012). Pressure driven digital logic in PDMS based microfluidic devices fabricated by multilayer soft lithography. Lab Chip.

[45] Knight J.B., Vishwanath A., Brody J.P., Austin R.H. (1998). Hydrodynamic focusing on a silicon chip: Mixing nanoliters in microseconds. Phys. Rev. Lett..

[46] Martel J.M., Toner M. (2014). Inertial focusing in microfluidics. Annu. Rev. Biomed. Eng..

[47] Xuan X., Zhu J., Church C. (2010). Particle focusing in microfluidic devices. Microfluid. Nanofluid..

[48] Aoki R., Yamada M., Yasuda M., Seki M. (2009). In-channel focusing of flowing microparticles utilizing hydrodynamic filtration_Supplementary material. Microfluid. Nanofluid..

[49] Gascoyne P.R.C., Vykoukal J. (2002). Review Particle separation by dielectrophoresis. Electrophoresis.

[50] Huang L.R., Cox E.C., Austin R.H., Sturm J.C. (2004). Lateral Displacement.

[51] Valero A., Braschler T., Demierre N., Renaud P. (2010). A miniaturized continuous dielectrophoretic cell sorter and its applications. Biomicrofluidics.

[52] Yamada M., Seki M. (2005). Hydrodynamic filtration for on-chip particle concentration and classification utilizing microfluidics. Lab Chip.

[53] Serex L., Braschler T., Filippova A., Rochat A., Béduer A., Bertsch A., Renaud P. (2018). Pore Size Manipulation in 3D Printed Cryogels Enables Selective Cell Seeding. Adv. Mater. Technol..

[54] Colosi C., Costantini M., Barbetta A., Dentini M. (2017). Microfluidic bioprinting of heterogeneous 3d tissue constructs. Methods Mol. Biol..

[55] Constantinescu V.N. (1995). Laminar Viscous Flow.

[56] Sideridou I.D., Achilias D.S., Karava O. (2006). Reactivity of benzoyl peroxide/amine system as an initiator for the free radical polymerization of dental and orthopaedic dimethacrylate monomers: Effect of the amine and monomer chemical structure. Macromolecules.

[57] Alsson B.E.O.P. (1997). Effective intercellular communication distances are determined by the relative time constants for cyto/chemokine secretion and diffusion. Engineering.

[58] Bianconi E., Piovesan A., Facchin F., Beraudi A., Casadei R., Frabetti F., Vitale L., Pelleri M.C., Tassani S., Piva F. (2013). An estimation of the number of cells in the human body. Ann. Hum. Biol..

